# Small field dosimetry for the small animal radiotherapy research platform (SARRP)

**DOI:** 10.1186/s13014-017-0936-3

**Published:** 2017-12-28

**Authors:** Mihaela Ghita, Stephen J. McMahon, Hannah F. Thompson, Conor K. McGarry, Raymond King, Sarah O. S. Osman, Jonathan L. Kane, Amanda Tulk, Giuseppe Schettino, Karl T. Butterworth, Alan R. Hounsell, Kevin M. Prise

**Affiliations:** 1 Centre for Cancer Research and Cell Biology, Queen’s University Belfast, 97 Lisburn Road, Belfast, BT9 7AE UK; 2 Radiotherapy Physics, Northern Ireland Cancer Centre, Belfast City Hospital, Belfast, BT9 7AB UK; 3 Xstrahl Inc, 480 Brogdon Road, Suite 300, Suwanee, GA 30024 USA; 4 Xstrahl Ltd, The Coliseum, Watchmoor Park, Riverside Way, Camberley, Surrey, GU15 3YL UK; 5 National Physical Laboratory, Hampton Road, Teddington, Middlesex, TW11 0LW UK

**Keywords:** Commissioning, Small animal radiotherapy research platform (SARRP), Preclinical radiotherapy, Radiobiology

## Abstract

**Background:**

Preclinical radiation biology has become increasingly sophisticated due to the implementation of advanced small animal image guided radiation platforms into laboratory investigation. These small animal radiotherapy devices enable state-of-the-art image guided therapy (IGRT) research to be performed by combining high-resolution cone beam computed tomography (CBCT) imaging with an isocentric irradiation system. Such platforms are capable of replicating modern clinical systems similar to those that integrate a linear accelerator with on-board CBCT image guidance.

**Methods:**

In this study, we present a dosimetric evaluation of the small animal radiotherapy research platform (SARRP, Xstrahl Inc.) focusing on small field dosimetry. Physical dosimetry was assessed using ion chamber for calibration and radiochromic film, investigating the impact of beam focus size on the dose rate output as well as beam characteristics (beam shape and penumbra). Two film analysis tools) have been used to assess the dose output using the 0.5 mm diameter aperture.

**Results:**

Good agreement (between 1.7–3%) was found between the measured physical doses and the data provided by Xstrahl for all apertures used. Furthermore, all small field dosimetry data are in good agreement for both film reading methods and with our Monte Carlo simulations for both focal spot sizes. Furthermore, the small focal spot has been shown to produce a more homogenous beam with more stable penumbra over time.

**Conclusions:**

FilmQA Pro is a suitable tool for small field dosimetry, with a sufficiently small sampling area (0.1 mm) to ensure an accurate measurement. The electron beam focus should be chosen with care as this can potentially impact on beam stability and reproducibility.

**Electronic supplementary material:**

The online version of this article (10.1186/s13014-017-0936-3) contains supplementary material, which is available to authorized users.

## Background

The development of advanced radiotherapy approaches in radiation oncology has been driven largely by significant achievements in engineering and physics [1]. However, biologically driven strategies in clinical practice have been far less substantial. This lack of progress is likely explained by the differences between clinical practice, animal models, and irradiation techniques used in the laboratory [2].

Whilst the insight gained using traditional radiobiological irradiation techniques has been very important in understanding fundamental biology, it might not be entirely relevant for modern radiotherapy delivery techniques [3]. Therefore, the development of dedicated small animal image guided irradiation devices has gained considerable attention from radiobiology labs to translate clinical irradiation technologies into preclinical settings. As in clinical radiotherapy, small animal irradiation involves extensive engineering challenges. To achieve clinically relevant data, clinically relevant biological research and quality assurance must be performed to ensure precision and accuracy.

To date, two commercially available small animal image guided micro-irradiators exist: SARRP (Xstrahl Inc., Swanee, GA, USA) and XRAD225Cx (PXI North Branford, CT, USA). Additionally, in-house small animal image guided systems have been developed [4, 5]. These irradiators add complexity to commissioning, dosimetry, and traceability outside of the commercially available devices. Commissioning procedures have been previously described either for SARRP in a bespoke water phantom [6], or for the XRAD225Cx small-field irradiator with specific dosimetry techniques such as ion chambers and Gafchromic film [7] as well multi-institutional studies for both platforms [8]. Both studies indicated EBT radiochromic film dosimetry for small fields as challenging but also feasible, and set the basis for preclinical dosimetry.

Preclinical dosimetry has gained considerable interest and high throughput approaches have also been considered. A recent study focused on the automation of film scanning, and analysis after irradiation in different beam configurations [9] while comparing that with Monte Carlo simulations of the specific source and beam geometry. This study found similarities between the high throughput scanning and the previously established film scanning method. Also, the empirical beam model was found to be a useful tool to predict film measurements percentage depth dose and profiles with sufficient accuracy. Most of these studies only report the findings for relatively large beam sizes (3 mm −10 mm) while the main challenge in pre-clinical dosimetry is represented by very small irradiation fields corresponding to specific small animal anatomy [10]. Another recent study focused on developing an analytic source model for dose calculations. The aim of this particular study was to introduce and demonstrate the viability of a analytical source model to further improve the collimator design or the dose calculation algorithm [11]. However, while comparing two models the study lacks validation from physical measurements using classic dosimetry methods.

Dosimetry procedures for small animal image guided micro-irradiators originate from the medical physics codes of practice used by clinical radiotherapy departments. These practices normally incorporate specific corrections for low energy beams and backscatter for broad field exposures. However, the most important difference between clinical and pre-clinical dosimetry are the utilized field sizes: while dosimetry for stereotactic small fields involves Gafchromic films and thermoluminescent detectors for areas under 0.8 × 0.8 cm^2^ [11], preclinical dosimetry employs even smaller fields.

Considering the increasing interest in the highly conformal high dose delivery in radiotherapy today, there is little preclinical data provided on the small (<3 mm) field dosimetry. While new techniques are being used to introduce tumour tracking and respiratory gating to preclinical research [12, 13], these will further add to the complexity of the small field irradiation making it a challenging aspect for both clinical, and more so, preclinical radiotherapy.

In radiobiology, very small and precise radiation beams (soft X-ray and charged particles) have long been used to deliver radiation to specific subcellular compartments [14, 15]. However, as dosimetry, these tools mostly use different particle counters to calculate the exact energy delivered to the targeted cells.

The present paper shows the full commissioning of our SARRP (220 kVp) [16], including physical, focusing on the 0.5 mm diameter apertures. The small size apertures are intended to be used for a very precise beam delivery. The present work aims to elucidate the specific technical aspects of the small beam use in preclinical radiobiology. The beam characterisation and determination of the absorbed dose has been performed according to the AAPM TG-61 code of practice [17].

## Methods

### Dosimetry and therapeutic beam calibration

Measurements from a Farmer® ionisation chamber Type 30,012 (PTW Freiburg) with a sensitive volume of 0.6 cm^3^, at 2 cm in water were used to calibrate EBT films. The irradiation time was determined based on the output from the Farmer® ionization chamber readings. EBT3 film calibration consisted of exposing single films to 5 different doses ranging from 0 to 9 Gy (0.5, 1, 2, 5 and 9 Gy) in an identical setup to the ionization chamber. Ionization chamber readings and a set of calibration films were taken at the beginning of each day for broad field calibration before the treatment beam was used.

A specifically designed solid water commissioning phantom was used to determine the absorbed dose at different depths as previously described [6]. All measurements were performed by irradiating the commissioning phantom on three independent occasions for each aperture size (10 × 10 mm, 5 × 5 mm, 3 × 3 mm, 3 × 9 mm and circular apertures with 1 and 0.5 mm diameter), and each Source to Surface Distance (SSD) (31, 34 and 38 cm). As previously shown, the optical density of the film changes with time after radiation exposure [18]. Therefore a set of calibration films was exposed prior to each set of measurements.

### Full therapeutic dose characterization with EBT film

Film was handled according to the procedures described in the (AAPM) Task Group 55 report, and cut at least 6 h before exposure to radiation [17]. Prior to irradiation, films were loaded in the commissioning phantom, consisting of 0.5 cm thick solid water slabs. Films were positioned at depths ranging from 0 mm to 70 mm between solid water blocks as previously described [6]. When measuring the dose depth profiles, film thickness was also considered, with the top film exposed at a depth of 0.15 mm, and the bottom film at a depth of 72.55 mm. One stack was irradiated for each aperture size and at 3 different SSDs. Each experiment was performed three times and the data presented as mean values ± standard error.

A large focal sport (5.5 mm) was employed for all apertures, with a maximum tube potential (220 kVp) and maximal tube current (13 mA). For 0.5 mm aperture, a small focal spot was also employed (1 mm) for the maximum tube potential and 3 mA. All film stacks were exposed from 90 s − 15 min to minimize the noise associated with the statistical errors due to low optical density of the films. For large apertures, an exposure time of 90 s was used, for 0.5 mm aperture with both focal spots, 15 min exposure time was used, as 90 s would not induce a quantifiable optical density change for small beams.

### Film analysis

Films were scanned using an EPSON V700 scanner set to professional mode without colour correction. A scanning resolution of 400 dpi was used for each of the collimators except for the 0.5 mm collimator which was scanned at a resolution of 600 dpi, with pixel sizes of 0.063 mm and 0.042 mm respectively. With all films, a non-irradiated film was also scanned to allow correction for background in the absence of radiation. All films were cut at least 6 h before exposure, and scanned at least 24 h after irradiation [18].

The exposed films were analyzed using Matlab codes previously described [6] and FilmQA Pro (Ashland Scientific) software. This uses multi-channel dosimetry to effectively separate out non-dose dependent abnormalities from the radiochromic film images. The process was shown to improve the integrity of the dose information by removing disturbances in the scanned images caused by non-homogeneity of the radiochromic film and artifacts caused by the scanner [18].

### Monte Carlo

To provide comparisons for the physical dosimetry, the SARRP X-ray source and collimation system were simulated in Geant4 v10.3.p02 [19]. These included simulation of the full geometry of the X-ray source target based on manufacturer specifications, and the physical collimator setup including the primary and secondary collimators, collimator support and final nozzle collimator, as described elsewhere [6]. The initial primary particles were monoenergetic 220 keV electrons fired along the central axis of the source towards the tungsten target. Beam divergence was modelled to produce appropriate physical focal spot sizes on the target by giving each electron a uniformly randomly sampled angular deviation from the primary beam direction, with the maximum deviation set to correspond to the manufacturer-reported spot diameter on the target.

These simulations made use of the Livermore low-energy physics lists throughout the simulation volume, with a 2 μm production cut applied to all particles, and 100:1 bremsstrahlung splitting to improve computational performance. To further reduce computation times associated with X-ray generation in this scenario, calculations were carried out recording the X-ray phase space in the final collimator support, just above the final adjustable collimator. This phase space was then used as input into a second simulation to determine the resulting dose-depth distribution for different collimators and different SDDs as appropriate. 1 × 10^9^ primary particles were simulated in both the X-ray phase space simulations, and the subsequent dose deposition calculations.

Target energy deposition was recorded in three dimensions throughout a 10 cm × 10 cm × 10 cm water phantom, whose upper surface was placed at the appropriate SSD from the electron beam spot on the target. Dose-depth curves were then calculated by scoring the total dose deposited in the central 0.25 mm radius section of the water phantom along the beam path. Energy deposition was also calculated across the whole area of the source at the surface and at a depth of 7.2 cm for comparison with experimental observations.

## Results

### Validation of manufacturer dosimetry

In order to determine the dose rate, as a function of depth, Gafchromic films were evaluated against the calibration curve obtained. The dose map for a set of films was acquired along with a specific dose at a chosen point and a beam profile for each aperture as shown in Additional file 1: Figure S1, Additional file 2: Figure S2, and Additional file 3: Figure S3.

**Fig. 1:**
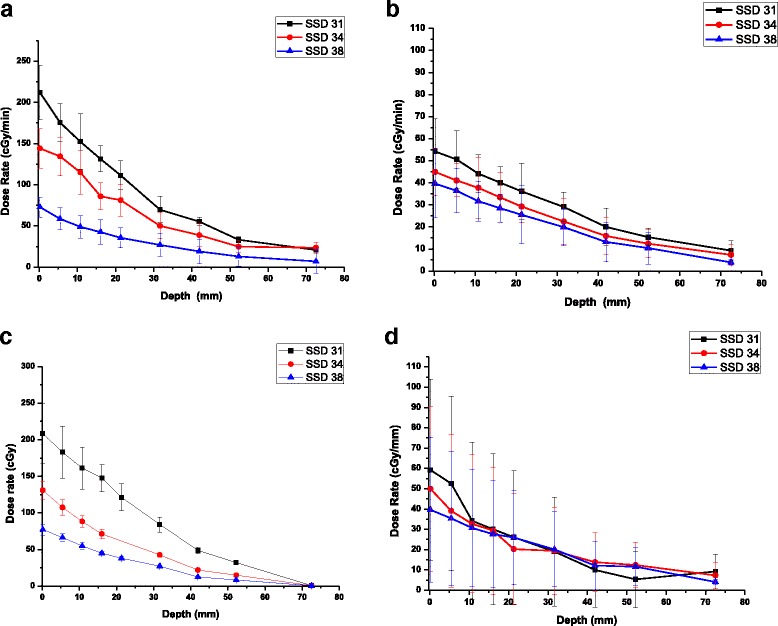
Depth dose deposition profile 0.5 mm apperture using a broad **a**) and **c**) and **a** fine focus **b**) and **d**) beam for the three SSDs:31 cm (black), 34 cm (red) and 38 cm (blue). Data was analysed and quantified using FilmQA software **a**) and **b**) and Matlab codes **c**) and **d**). Data represents the average ± standard error (*n* = 3)

As previously observed [6] the output from the 0.5 mm apertures dependends on the beam spot-size and fluence distribution. To further investigate this effect, the dose depth profiles were measured for the 0.5 mm diameter aperture for both a large and a small focal spot. FilmQA Pro measurements are presented in Fig. 1a and b for bright and fine focus, respectively. As a comparison, Xstrahl dosimetry data using methods previously described [6] is presented in Fig. 1c and d for the same focal spot sizes.

The most important difference between the two focal spots used are the sharp drop in dose rate: from 210 cGy/min when employing the bright focus at 220 kV and 13 mA, to 55 cGy/min for a small focus and 220 kV and 3 mA configuration. Concurrently, the efficiency increases slightly from 16 cGy/min/mA to 18.3 cGy/min/mA for large and small focal spot respectively. Another observation is related to the sampling area used when measuring the dose measurement. While the Matlab codes (1c, 1d) measure the optical density over an area of 2.5 mm^2^, FilmQA Pro uses a 0.5 mm radius sampling as the smallest measured area (1a, 1b). While this smaller error bars, it more accurately reflects the structure of the X-ray beam, which is very important when measuring the dose output from small radiation fields.

The dose depth profiles for the entire range of apertures and the three different SSDs are presented in Additional file 1: Figure S1. The data is in good agreement with the dosimetry provided by the manufacturer (data not shown).

### Focal spot choice

To quantify the beam shape and uniformity, beam profiles were generated for both focal spots involved using the FilmQA Pro software.

**Fig. 2:**
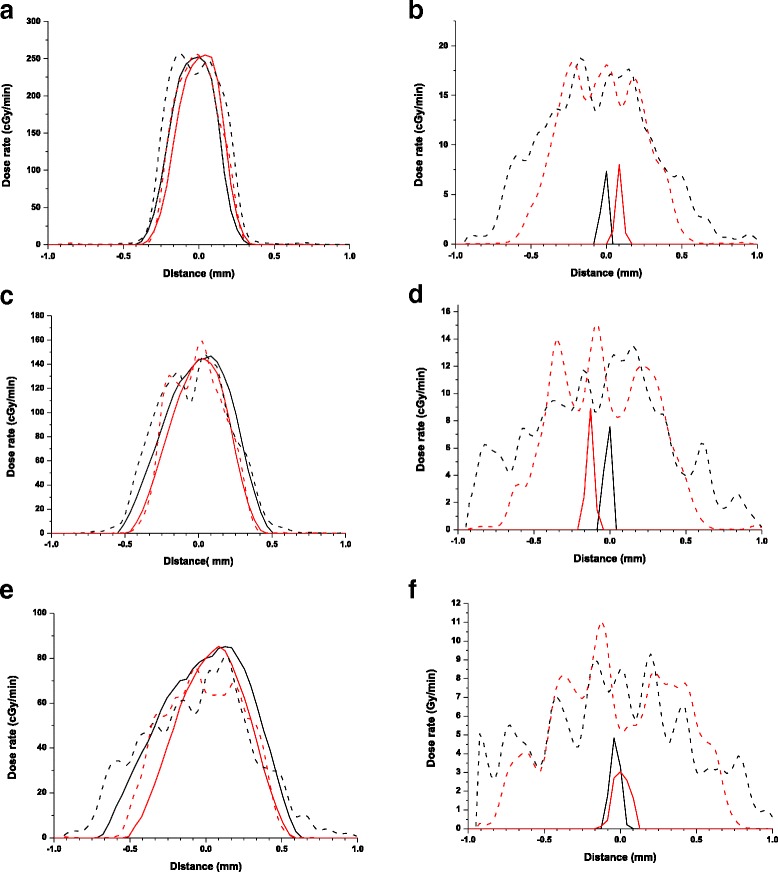
Beam uniformity across the irradiated area using a 0.5 mm diameter aperture and a broad focus at: 31 cm SSD **a**) and **b**), 34 cm SSD **c**) and **d**), and 38 cm SSD **e**) and **f**). Depths of 0.15 mm: **a**),**c**),**e**), and 72.55 mm: **b**),**d**),**f**) are shown. Measured profiles along x axis are black solid lines, and profiles along y axis are shown in red solid lines. Monte Carlo calculated beam profiles are dashed lines (black – x axis and red- y axis)

The 0.5 mm aperture beam profiles are presented for the three SSDs (31, 34, and 38 cm) for bright focus at 0.15 mm depth in Fig. 2, panels a, c and e, along both x and y axis. The large focus beam profiles show a small beam asymmetry along the x (black) and y (red) axes for the entrance beams. As expected, this is intensified at a depth of 72.25 mm as seen in b, d and f panels.

**Fig. 3:**
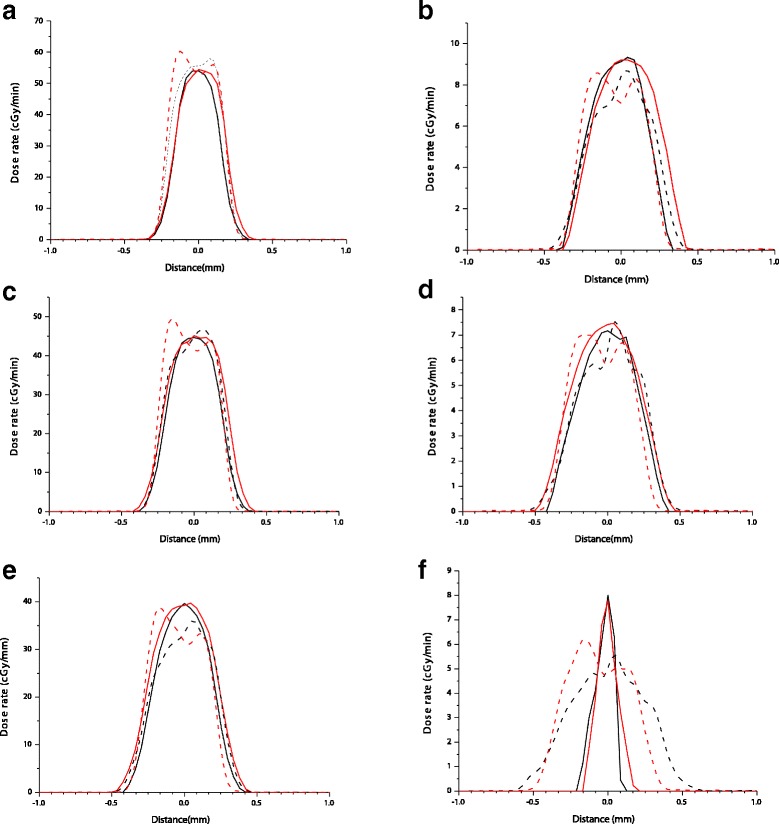
Beam uniformity across the irradiated area using a 0.5 mm diameter aperture and a fine focus at: 31 cm SSD for **a**) and **b**), 34 cm SSD **c**) and **d**), and 38 cm SSD **e**) and **f**) . Depths of 0.15 mm **a**),**c**),**e**), and 72.55 mm b),d),f) are shown. Measured profiles along x axis are black solid lines, and profiles along y axis are shown in red solid lines. Monte Carlo calculated beam profiles are dashed lines (black – x axis and red- y axis)

Beam profiles for the 0.5 mm aperture and a small focus in the same conditions are shown in Fig. 3 with panels a, c, and e show the entrance beam profile (Additional file 2). Panels b, d and f show the beam profiles at a 72.25 mm depth. Compared to Fig. 2, the beam symmetry is significantly improved, especially for the 72.25 mm depth.

In addition to the measured profiles, Monte Carlo modelled profiles are also presented for entrance doses profiles in Figs. 2 and 3. There is a considerable level of statistical uncertainty in these profiles due to the small volumes involved in scoring these profiles and the limited sampling of the primary photon space. Despite this, it can be seen that the overall trend in beam profile is well reproduced, producing reasonable estimates for spot diameter and spread, including the increasing heterogeneity and spread with the broad focus compared to the fine focus, suggesting that this model broadly reflects the source of these trends in spot size. Some of the remaining disagreement in these observations may be due to the limited electron beam model, which only considers a simple radially symmetric source, rather than a more detailed beam model [10].

In addition, these observations are confounded at the greatest depths due to the limitations of the clinical film scoring technique. As the beam spot at such depths is faint and surrounded by an elevated background due to scattering, the software algorithm has limited capability to detect it. Instead, in many cases it only detects a single central beam point with confidence, giving the appearance of increased beam sharpness. This is particularly apparent when compared to the Monte Carlo calculated profiles which present a much broader and more complex beam shape at these depths, although this comparison is further complicated due to the high level of statistical noise in these points (Fig. 2). These observations highlight the need for the use of appropriate measurement techniques.

**Fig. 4:**
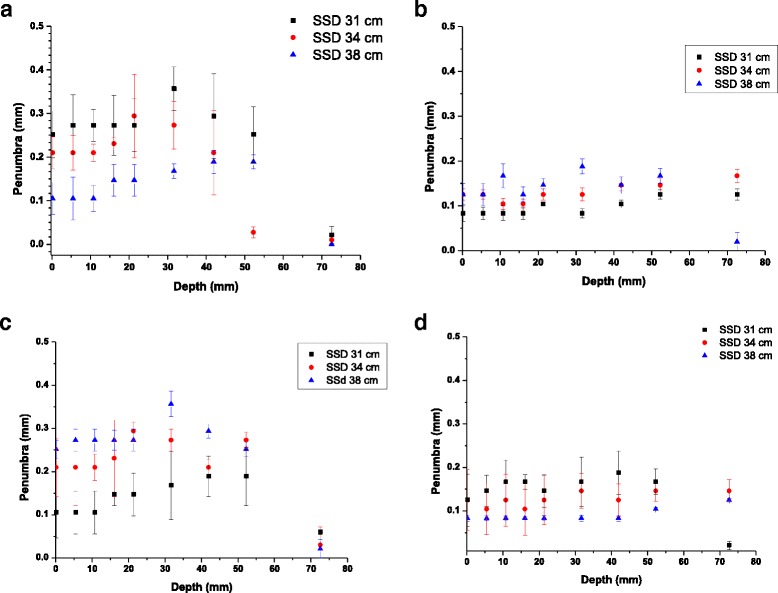
Beam Penumbra presented after 3 independent repeats at different times. FilmQA Pro data is presented for bright focus **a**) and fine focus **b**). Manufacturer data is presented for bright focus **c**) and fine focus **d**). Data presented represents an average ± standard error (n = 3)

Beam penumbra, calculated as the distance from the point of 50% of the maximum dose to the last reading on the film was also measured for both focal spot sizes and presented in Fig. 4. The data is presented for the entire depth of the phantom, and, for panels a and b, after 3 independent measurements. The difference between the error bars between the four panels is again due to the sampling size during the measurements. The high variation in the beam shape and penumbrae observed when using the broad and fine focus is also shown in Fig. 4 as a result of both film reading methods: Fig. 4a and b are FilmQA Pro readings for 3 independent repeats, while Fig. 4c and d are Matlab measurements. While FilmQA Pro is a tool used in clinic, previous studies report on using this software for preclinical dosimetry [20]. However, no studies have used this approach for small field dosimetry. Since this was a measurement performed over different runs, the large error bars for the large beam focus emphasize the poor beam stability over time.

**Fig. 5:**
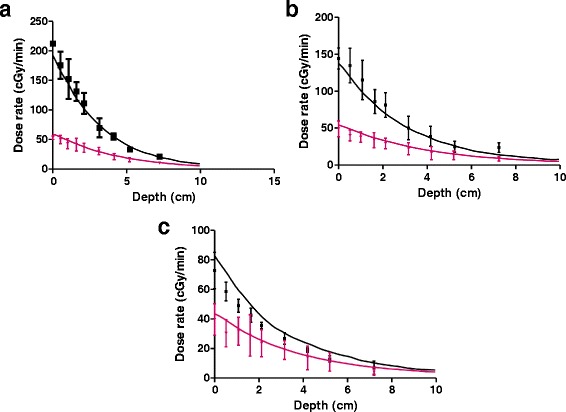
Monte Carlo simulations (solid lines) for bright focus (black) and fine focus (red) as compared with measured data (symbols) for **a**) 31 cm SSD, **b**)34 cm SSD and **c**) 38 cm SSD

A good agreement was found between measured data and the Monte Carlo simulations carried out for both large and small focal spots, within experimental uncertainties (Fig. 5). Further testing and validation of Monte Carlo models of SARRP dosimetry may prove to be a useful tool in SARRP planning and verification.

## Discussion

The discipline of translational preclinical radiotherapy has been enabled through the emergence of small animal image guided micro-irradiation platforms. These systems have significant potential to improve the impact of transitional radiobiology studies [21].

However, as with clinical treatments, irradiation of small animals should also be subject to strict quality assurance protocols ensuring robust dosimetry, and dose verification standards are followed. Strict dosimetry protocols will provide to be vital for animal welfare and further minimize the number of animals required for a study to ensure a good power is obtained from the study. Based on the power calculations for a study with two experimental arms, a 20% variation in radiation response and a typical dose uncertainty of 10% in small animal IGRT. This leads to a sample size of 23 animals required to reduce the dose uncertainty to 1% (considering 80% power with a 5% significance level for 2-tailed t-test). In this context, a modest 5% reduction in dose uncertainty would significantly decrease the sample size to only 10 animals.

Previous work has described a standard procedure for small field dosimetry on SARRP [6], and served as a basis of the current study, and initial commissioning on our system. In the current study, doses in air and a solid water phantom were measured and cross calibrated with EBT3 films. This study also states the need for an alternative strategy for the use of 0.5 mm aperture, as using the standard therapeutic beam parameters leads to a suboptimal beam quality.

While the use of small beams in pre-clinical scenario have several challenges including organ movement and dosimetry, the increased use of stereotactic radiosurgery clinically demands a greater input from pre-clinical tests. This is a potentially new avenue for future preclinical studies. Small field depth dose profiles for different electron beam focus sizes show dramatic differences for the 0.5 mm diameter aperture for all SSDs. While a bright focus (the recommended therapeutic setup for SARRP) will ensure a lower delivery time for all a 0.5 mm aperture, it is significantly smaller than the beam spot, causing significant beam heterogeneities, particularly at greater depths. These are challenging to include in planning and may potentially lead to very large errors in delivery. This is highlighted in the independent beam penumbra measurements shown in Fig 4. Here, the broad focus measurements showed variation between different days and corresponding large uncertainties, while the small spot size proved much more stable.

Beam penumbra becomes increasingly important when employing a very small field. Since a high uncertainty in the field edges are associated with inaccuracies in beam positioning as well as dose delivery. The width of the penumbra regions are largely dependent on the scattering from the collimator system in this case. These observations indicate the small focus configuration more suitable for pre-clinical small field irradiations. Measurement of these effects must be handled with care, however, as the low spot intensity and increasing penumbra may cause tools optimised for alternative applications such as the larger fields used clinically to fail to produce meaningful results.

Precise small animal irradiators (such as the SARRP used in this study) are a technology that can revolutionize the field of radiobiology. Their multi-disciplinary relevance to radiobiology has the potential to offer numerous avenues of preclinical investigation [3]. However, this potential impact of technology may be limited due to poorly defined dosimetry standards. It is essential that dosimetry and QA techniques are well specified and implemented across different sites. These practices will help improve reproducibility and allow accurate comparison of radiobiological data from different investigators. In this way, uncertainty in dose can be removed as a confounding factor in preclinical radiobiology investigations and small animal image guided micro-irradiators can be used to their fullest potential.

This study highlights that, when preclinical stereotactic irradiation fields are used, a practical compromise needs to be considered when deciding the treatment beam configuration used. While a small focus will involve a significantly smaller dose rate and therefore a higher overall treatment delivery time, it also ensures a more stable and homogenous beam. For the 0.5 mm aperture a large focal spot size will deliver 210 cGy/min, however the beam heterogeneity, penumbra and poor stability will potentially affect the statistical power of the study.

## Conclusions

The technological evolution from simple, broad field irradiation configurations, to more sophisticated dose deliveries for preclinical radiobiology experiments has introduced new dosimetry challenges for preclinical research. Robust QA and dosimetry techniques are a key part of using novel treatment platforms using very small irradiation fields. This study establishes FilmQA Pro as a suitable tool to perform small field measurements, with a higher accuracy of the measurements. Furthermore, the electron beam focus should be chosen with care as this can impact on beam stability and reproducibility.

## Additional files

Additional file 1: Figure S1. Profiles for Dose Deposition for the entire range of therapeutic apertures for broad focus irradiation. Data was obtained using FilmQA Pro for 3 independent exposures for a) 31 cm SSD, b) 34 cm SSD and c) 38 cm SSD. Data is shown as average from 3 independent repeats ± standard error. (PDF 314 kb)

Additional file 2: Figure S2. Beam uniformity profile across the irradiated area for full set of therapeutic apertures. Beam profiles are presented for three different SSD at a depth of 0.15 mm in the phantom. Apertures sizes are: a) 5 × 5 mm, b) 3 × 3 mm, c) 3x9mm along x axis, d) 3x9mm along y axis, e) 1 mm diameter and f) 0.5 mm diameter. (PDF 317 kb)

Additional file 3: Figure S3. Beam uniformity profile across the irradiated area for full set of therapeutic apertures. Beam profiles are presented for three different SSD at a depth of 0.15 mm in the phantom. Apertures sizes are: a) 5 × 5 mm, b) 3 × 3 mm, c) 3x9mm along x axis, d) 3x9mm along y axis, e) 1 mm diameter and f) 0.5 mm diameter. (PDF 326 kb)

## Abbreviations

AAPM: The American Association of Physicists in Medicine
CBCT: Cone Beam Computed Tomography
ICRU: International Commission on Radiation Units
IGRT: Image Guided Radiotherapy
NIST: National Institute of Standards and Technology
NPL: National Physical Laboratory
SARRP: Small Animal Radiation Research Platform
SSD: Source to Surface Distance
