# Supplementary figures and images for: Small field dosimetry for the small animal radiotherapy research platform (SARRP)
Source: Radiat Oncol. 2017 Dec 28;12:204. doi: 10.1186/s13014-017-0936-3 (PMC5745702; doi:10.1186/s13014-017-0936-3)

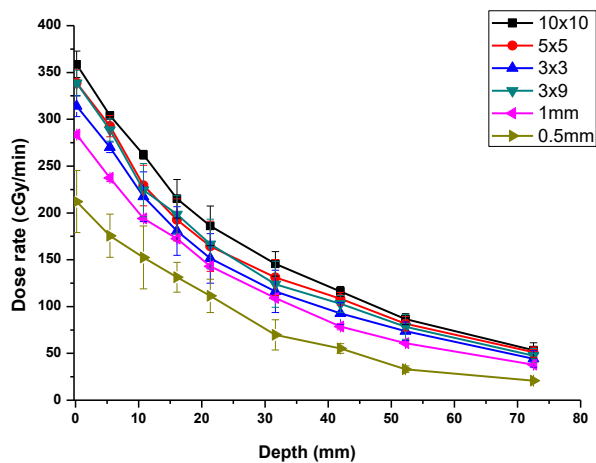

a)

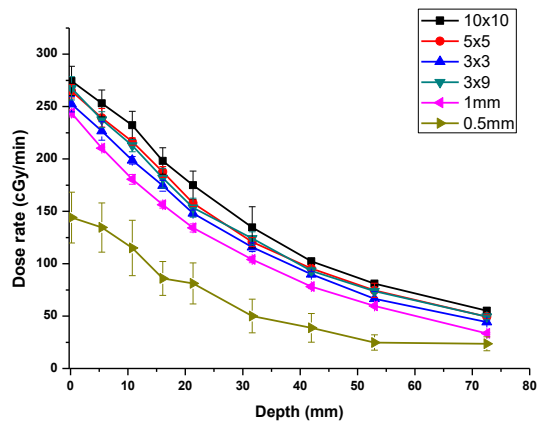

b)

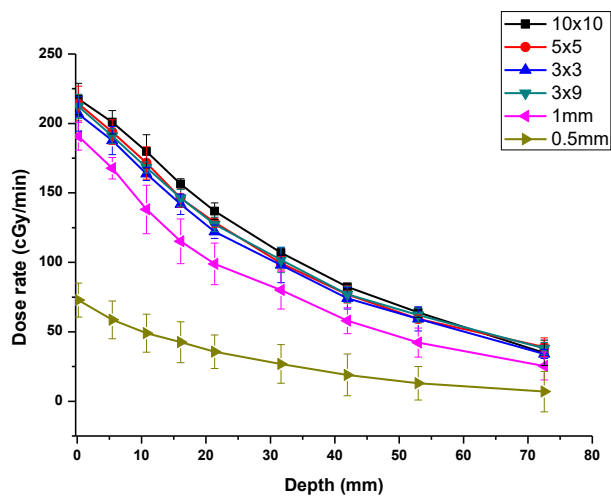

c)

Supplement: Supplementary file 1 — Profiles for Dose Deposition for the entire range of therapeutic apertures for broad focus irradiation. Data was obtained using FilmQA Pro for 3 independent exposures for a) 31 cm SSD, b) 34 cm SSD and c) 38 cm SSD. Data is shown as average from 3 independent repeats ± standard error. (PDF 314 kb) [file 13014_2017_936_MOESM1_ESM.pdf]

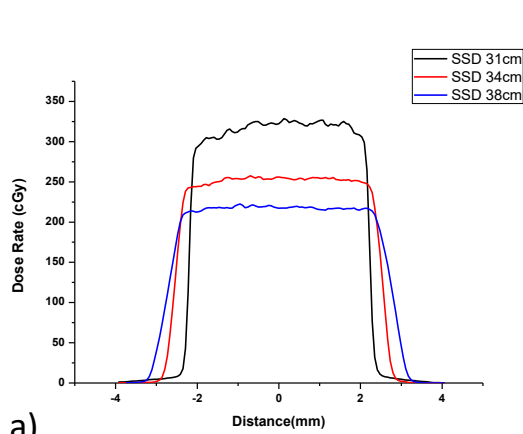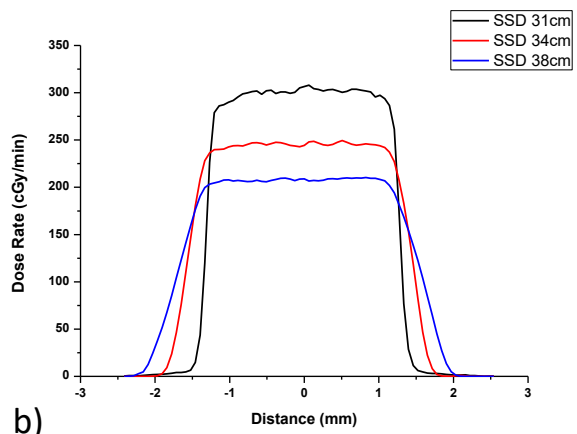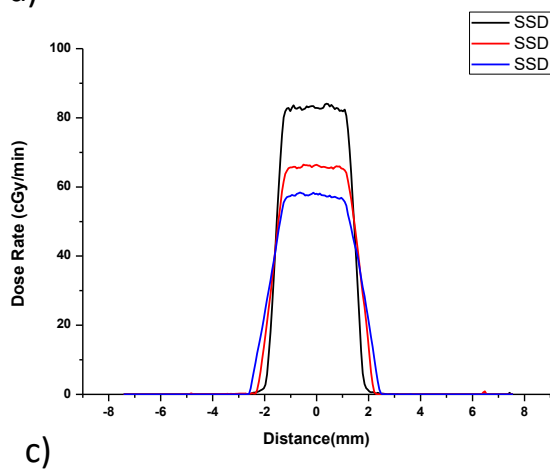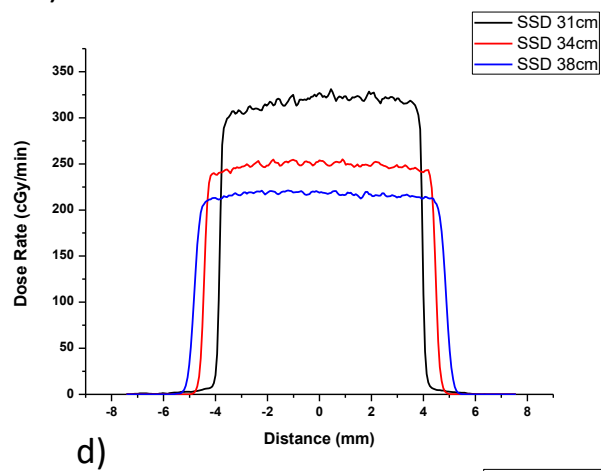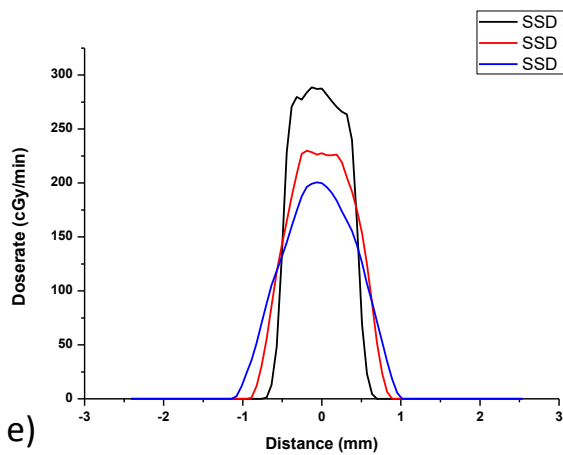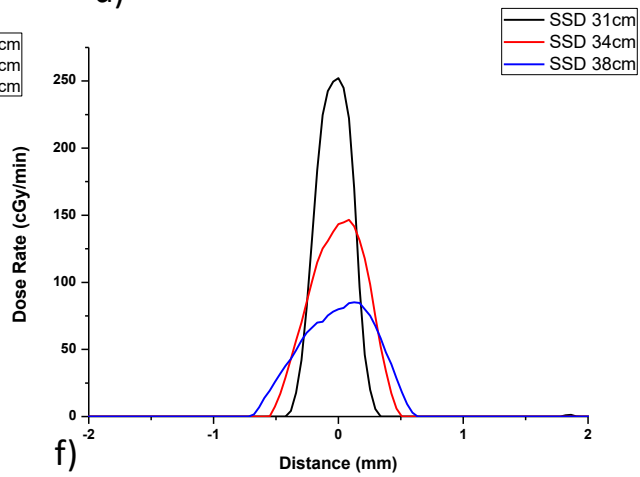

Supplement: Supplementary file 2 — Beam uniformity profile across the irradiated area for full set of therapeutic apertures. Beam profiles are presented for three different SSD at a depth of 0.15 mm in the phantom. Apertures sizes are: a) 5 × 5 mm, b) 3 × 3 mm, c) 3x9mm along x axis, d) 3x9mm along y axis, e) 1 mm diameter and f) 0.5 mm diameter. (PDF 317 kb) [file 13014_2017_936_MOESM2_ESM.pdf]

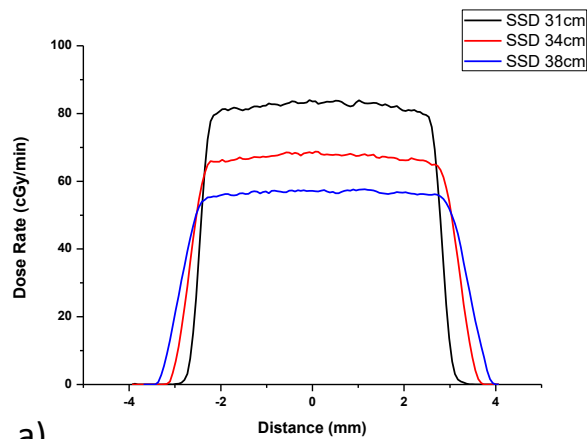

a)

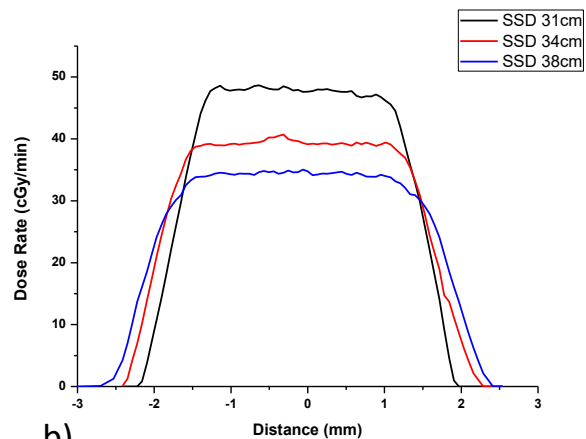

b)

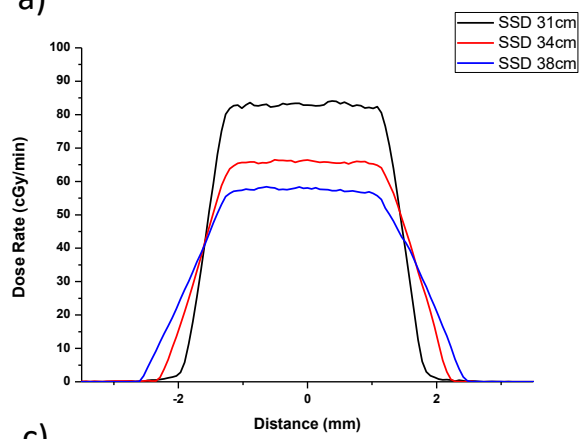

c)

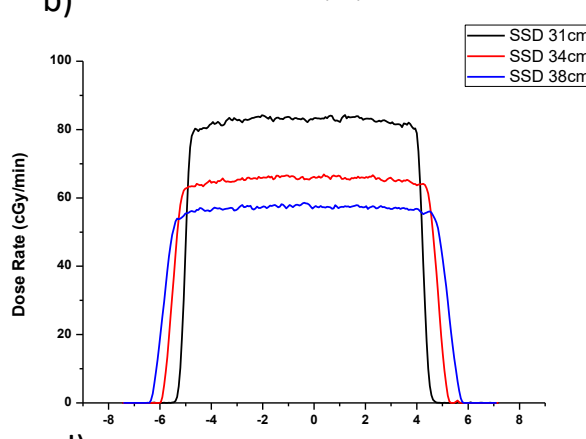

d)

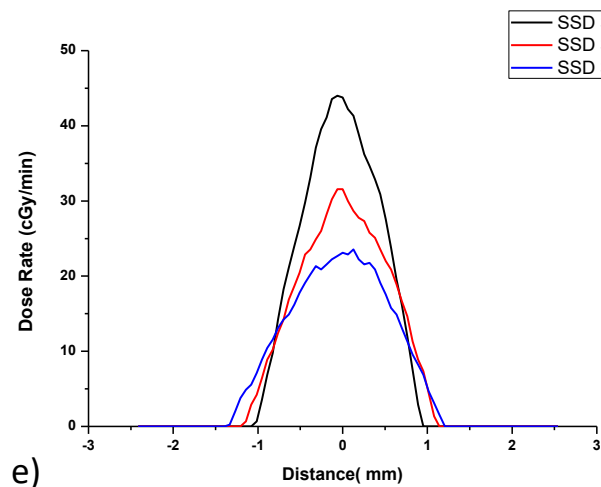

e)

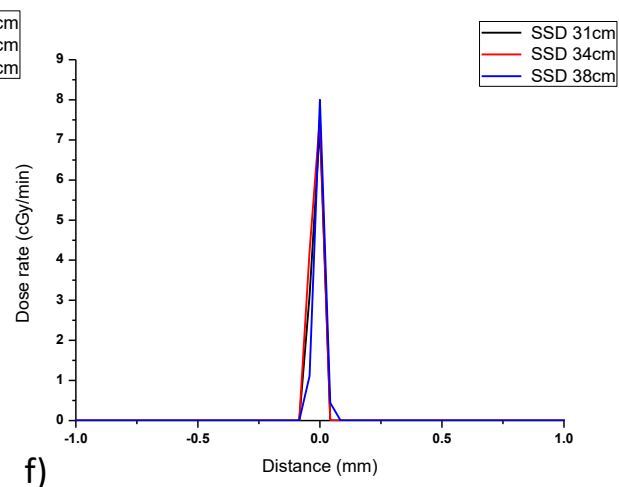

f)

Supplement: Supplementary file 3 — Beam uniformity profile across the irradiated area for full set of therapeutic apertures. Beam profiles are presented for three different SSD at a depth of 0.15 mm in the phantom. Apertures sizes are: a) 5 × 5 mm, b) 3 × 3 mm, c) 3x9mm along x axis, d) 3x9mm along y axis, e) 1 mm diameter and f) 0.5 mm diameter. (PDF 326 kb) [file 13014_2017_936_MOESM3_ESM.pdf]
